# Isolated Lung Metastasis Prostate Cancer

**DOI:** 10.1097/MS9.0000000000003204

**Published:** 2025-03-18

**Authors:** Emmanuel Mduma, Hellen Makwani, Said Msuya, Eligi D. Shao, Elijah Ussiri, James Kitinya

**Affiliations:** a Department of Clinical Oncology, Rabininsia Memorial Hospital, Dar es Salaam, Tanzania; b Department of Radiology, Rabininsia Memorial Hospital, Dar es Salaam, Tanzania; c Department of Surgery, Rabininsia Memorial Hospital, Dar es Salaam, Tanzania; d Department of Anatomical Pathology, Muhimbili University of Health and Allied Sciences, Dar es Salaam, Tanzania; e Mutokiti diagnostic laboratories, Dar es Salaam, Tanzania

**Keywords:** lung metastasis, palliative care, pathology, prostate cancer

## Abstract

**Introduction and importance:**

Prostate cancer is the second most diagnosed malignancy (after lung cancer) in men worldwide. The most common site for metastasis of prostate cancer is bone (84%), followed by distant lymph nodes (10.6%), liver (10.2%), and lung being the least with 9.1%. Isolated lung metastasis is very rare and is present in less than 4.6% of metastatic prostate cancer. This is the first case of de novo isolated lung metastasis of prostate cancer in our setting.

**Case presentation:**

We report a case of an 81-year-old male of African ethnicity who was diagnosed with isolated lung metastasis prostate adenocarcinoma. The total prostate specific antigen at the time of diagnosis was 182 ng/ml and grade group 3.

**Clinical discussion:**

The prognosis of prostate cancer patients with pulmonary metastasis is reported to be limited. Prostate cancer patients with lung metastasis have median overall survival of approximately 19 months.

**Conclusion:**

Although isolated pulmonary metastasis is rare in prostate cancer, it should not be excluded especially in patients with perineural invasion; however, further investigations are required to exclude metastasis to other sites.

## Introduction

Prostate cancer (PCa) is a malignancy arises from prostate gland with high prevalence to men above 50 years of age^[^1^]^. It ranks as the second most frequently diagnosed cancer (following lung cancer) in men globally, with 1 467 854 new diagnoses and 397 430 fatalities reported in 2020^[^2^]^. The occurrence of cases and fatalities is linked to advancing age, with the average age at the point of diagnosis being 66 years. The incidence is higher in men of African descent than in Caucasian men^[^3^]^, and in Tanzania, where it is the second most frequently occurring cancer (10.7%), it is the leading cancer in men (26.4%)^[^2^]^.

PCa commonly metastasizes to the bone (84%), distant lymph nodes (10.6%), liver (10.2%), and thorax (9.1%)^[^4^]^ but rarely to isolated lungs^[^5^]^.

The most common PCa pathology is adenocarcinoma (93.7%); other histology types can be sarcoma, neuroendocrine and small cell PCa^[^6^]^. PCa frequently exhibits perineural invasion (PNI), which is seen in 11% to 38% of samples and is one of the common indications for metastasis^[^7^]^. About 84% of metastasis PCa presents with PNI^[^7^]^.

The survival rate is high for early PCa; 5-year survival is more than 99% for local disease, 32% for distant metastasis^[^8^]^, and only about 17% for lung metastasis^[^9^]^.

We report a case of an 81-year-old male of African ethnicity who was diagnosed with isolated lung metastasis prostate adenocarcinoma. This is the first case to be reported in Tanzania.

## Case presentation

**Figure 1.**
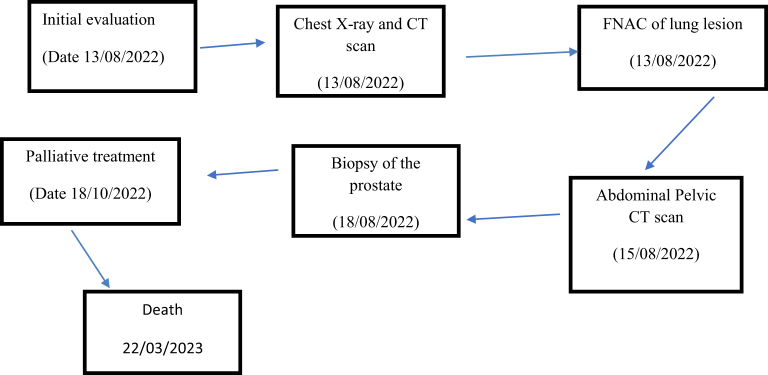
Time line of important events.

An 81-year-old male of African ethnicity, who was hypertensive for 5 years and on regular medication reported to our center (Fig. 1) with the complaint of a non-productive cough for 2 months associated with right-sided chest pain, chest tightness, and shortness of breath on activity (exertion dyspnea). He also had a history of increased urinary frequency, urgency, nocturia, and poor streaming for 3 months. He had no history of skeletal pain. ECOG—2, and on respiratory examination, there was reduced air entry on the right side of the chest, one-third of the lower chest, with a stony dull percussion note. Digital rectal examination showed normal anal verge and mucosa with free rectum, but the prostate was hard and had irregular margins.

**Figure 2.**
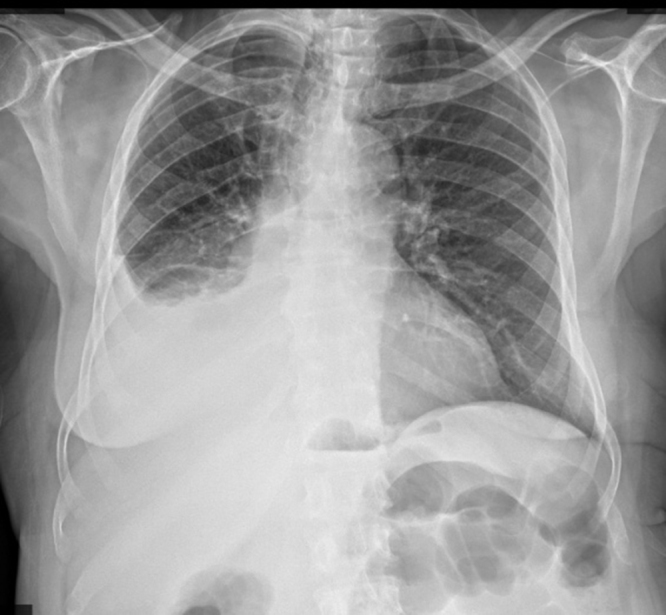
Chest X-ray in posterior aterior view. Homogenous opacity obliterating the right costophrenic angle with reduced right lung volume on the right hemi-thorax.

**Figures 3a and b:**
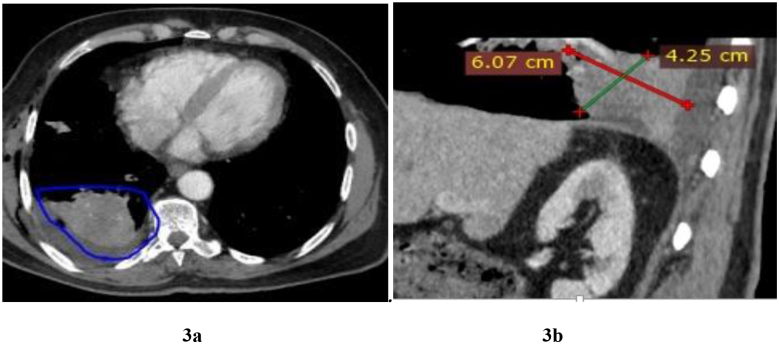
Chest CT scan; Axial and sagittal reformatted sections of chest at the level of the consolidation. Right lower lobe posterior basal segment consolidation (6.07 × 4.25) cm with no air broncograms associated with mild right pleural effusion.

**Figure 4.**
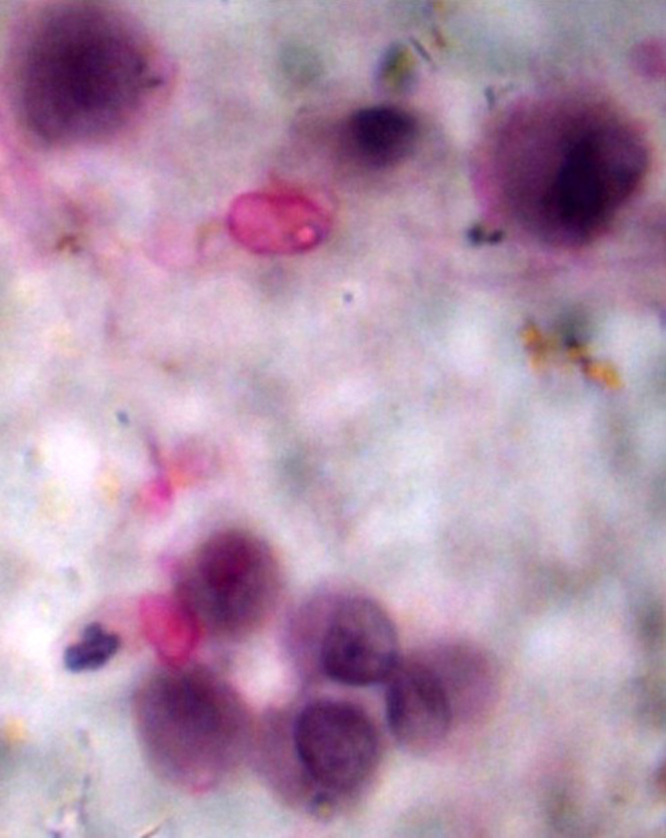
Large pleomorphic cells with abundant cytoplasm, few mesothelial cells, and lymphocytes.

**Figure 5.**
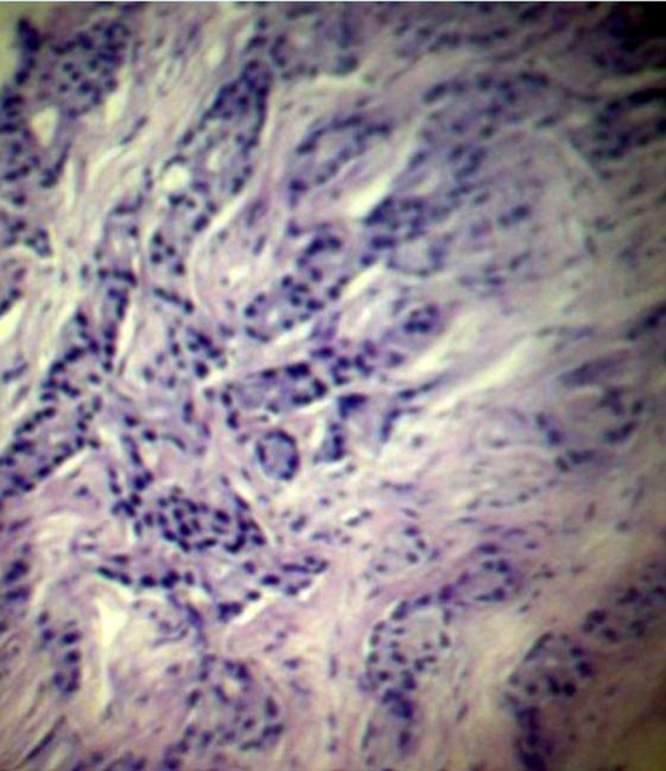
Infiltrates of atypical poorly formed and fused glands forming nests with peri neural invasion.

Laboratory investigations were done, including total prostate-specific antigen (PSA), which was 182 ng/ml (highly elevated); alkaline phosphatase, 92.4 U/L (normal); serum calcium, 2.07 mmol/l (normal); and complete blood count (WBC, 4.88 × 10^9^; Hb, 9.0 g/dl; and platelet, 168 × 10^9^). Radiological investigation was done (Fig. 1), including an X-ray of the chest that revealed a right-sided pleural effusion and decreased lung volume (Fig. 2). CT scan of the chest showed right lower lobe posterior basal segment consolidation (6.07 × 4.25) cm with no air broncograms associated with mild right pleural effusion. There were no associated satellite lesions or mediastinal lymph node enlargement; the conclusion was right posterior basal segmental collapse with a differential diagnosis of carcinoma (Figure 3a and b). CT-guided fine needle aspiration for cytology (FNAC) of the lung lesion showed large pleomorphic cells with abundant cytoplasm and few mesothelial cells and lymphocytes, which concluded metastatic adenocarcinoma of the prostate to the lungs (Fig. 4). Abdominal pelvic CT scan shows a left simple (Bosniac I) superior renal pole cyst, thoracic and lumbar spondylitis, and normal prostate size of 33 cc/grams with intraparenchymal calcification and nodulations. Trucut biopsy (Fig. 1) of the prostate showed all cylinders with an infiltrate of atypical cells forming glands, tubules, and nests with PNI, concluding prostate adenocarcinoma group 3 (Fig. 5). There was no bone involvement on bone scan imaging. At this point, the diagnosis of lung metastasis, AJCC 8th edition, stage IVB prostate adenocarcinoma was made.

He was treated with palliative intent with androgen deprivation therapy (ADT), given Goserelin 10.8 mg SC three monthly, Bicalutamide 50 mg PO OD for 1 month, together with docetaxel 120 mg IV 3 weekly for six cycles. Pleurodesis with bleomycin 60 IU was done to control pleural effusion and morphine syrup together with non-steroidal anti-inflammatory drug (NSAIDs) was given for pain management. Total PSA become 12.3 ng/ml from 182 ng/ml post 6 months of treatment. He survived 7 months post-diagnosis (Fig. 1), and the possible cause of death is acute respiratory distress with advanced PCa as the underlying cause.

## Clinical discussion

We report a case of 81-year-old male of African ethnicity who has been diagnosed with isolated lung metastasis prostate adenocarcinoma. Isolated lung metastasis PCa is rare, accounting for 1–4.6% of metastatic PCa cases^[^5,10^]^. This is the first documented case to be reported in Tanzania.

PCa is the prevalent malignancy in elderly males and one of the main causes of death for men globally^[^2^]^. PCa can metastasize to bone, lymph nodes, liver, and lungs. Some terminally ill patients with multiple metastases of PCa have lung metastases; however, isolated lung metastases without contemporaneous bone or lymph node metastases are extremely rare^[^10^]^. In this current case, right lower lobe posterior basal segment consolidation (6.07 × 4.25) cm with no air broncograms associated with mild right pleural effusion was reported from chest CT scan. FNAC of the lung lesion concludes metastatic adenocarcinoma of the prostate to the lungs. Apart from pulmonary site, there was no other organ with metastatic disease. The detection of metastatic and loco-regional disease has improved since the advent of Prostate- Specific Membrane Antigen Positron Emission Tomography - Computed Tomography (PSMA PET-CT)^[^11^]^. Tomography and bone scintigraphy are still used as standards for metastatic workups in Tanzania because PSMA PET-CT is not yet available in the country.

The Trucut biopsy of the prostate for this reported case showed all cylinders with an infiltrate of atypical cells forming glands tubules and nests with PNI. PNI is a common indication of tumor metastasis that can be detected in multiple malignancies, including PCa^[^12^]^. When PNI develops, the perineural niche is formed by the close interaction of tumor cells with the nerve cells in the tumor microenvironment, which gives the nerve cells a favorable environment for their survival and invasion^[^7,12^]^. The prevalence of PNI in PCa can be up to 44% and it is associated with high Gleason score (GS), angiogenesis, tumor invasion, and poor outcomes^[^13^]^. The GS of the current presented case was 4 + 3, grade group 3.

The treatment of metastatic high burden PCa is palliation. ADT for up to 3 years and Docetaxel 3 weekly for 6 cycles are given as the first-line treatment^[^14,15^]^. Radiation Therapy can be given to the pelvis and primary for low-burden metastasis disease as it improves failure-free survival outcome^[^16^]^. Other palliative care can include pain management, and other supportive treatment to improve quality of life. In this reported case, ADT together with Docetaxel 120 mg I.V 3 weekly for six cycles was given. Pleurodesis with Bleomycin 60 IU was given to control pleural effusion, and morphine syrup together with NSAIDS was given for pain management. Lutetium-177-prostate-specific membrane antigen-617, in conjunction with standard care treatment, has recently demonstrated an improvement in overall survival among men with advanced-stage prostate-specific membrane antigen-positive metastatic castration-resistant PCa^[^17^]^; however, this was not given to our patient due to limited resources. There are some documented cases of solitary lung metastasis from primary PCa with normal PSA levels^[^18^]^; the total PSA of our presented case was significantly high (182 ng/ml) at the time of diagnosis. The prognosis of PCa patients with pulmonary metastasis is reported to be limited^[^19^]^. PCa patients with lung metastasis has median overall survival of approximately 19 months^[^19^]^. The current reported case survived 7 months post diagnosis.

## Conclusion

Although isolated pulmonary metastasis is rare in PCa, it should not be excluded especially in patients with PNI; however, further investigations are required to exclude metastasis to other sites. In spite of limited prognosis for lung metastasis PCa; good palliative care should be maintained to improve the quality of life.

## Data Availability

All data that support the findings of this study are included in this article and its online supplementary material files. Further inquiries can be directed to the corresponding author.
